# Surgical treatment of a rare primary renal carcinoid tumor with liver metastasis

**DOI:** 10.1186/1477-7819-6-41

**Published:** 2008-04-22

**Authors:** Roberto Gedaly, Hoonbae Jeon, Thomas D Johnston, Patrick P McHugh, Randall G Rowland, Dinesh Ranjan

**Affiliations:** 1 Division of Transplantation and Hepatobiliary Surgery, Department of Surgery, University of Kentucky Medical Center, Lexington, Kentucky, USA; 2 Division of Urology, Department of Surgery, University of Kentucky Medical Center, Lexington, Kentucky, USA

## Abstract

**Background:**

Carcinoid tumors are characteristically low grade malignant neoplasms with neuroendocrine differentiation that arise in various body sites, most commonly the lung and gastrointestinal tract, but less frequently the kidneys, breasts, ovaries, testes, prostate and other locations. We report a case of a carcinoid of renal origin with synchronous single liver metastases on radiological studies.

**Case presentation:**

A 45 year-old patient who presented with abdominal pain was found on CT scan to have lesions in the right ovary, right kidney, and left hepatic lobe. CA-125, CEA, and CA 19-9 were within normal limits, as were preoperative liver function tests and renal function. Biopsy of the liver mass demonstrated metastatic neuroendocrine tumor. At laparotomy, the patient underwent total abdominal hysterectomy with bilateral salpingo-oophorectomy, radical right nephrectomy with lymphadenectomy, and left hepatectomy. Pathology evaluation reported a right ovarian borderline serous tumor, well-differentiated neuroendocrine carcinoma of the kidney (carcinoid) with 2 positive retroperitoneal lymph nodes, and a single liver metastasis. Immunohistochemistry revealed that this lesion was positive for synaptophysin and CD56, but negative for chromogranin as well as CD10, CD7, and CD20, consistent with a well-differentiated neuroendocrine tumor. She is doing well one year after her initial surgery, with no evidence of tumor recurrence.

**Conclusion:**

Early surgical intervention, together with careful surveillance and follow-up, can achieve successful long-term outcomes in patients with this rare malignancy.

## Background

Neuroendocrine carcinomas may originate in a wide variety of tissues and organs, including those that do not normally contain neuroendocrine cells [1]. These tumors may occur in pure forms or in association with conventional adenocarcinomas or squamous cell carcinomas [2]. Neuroendocrine tumors of the kidney include carcinoids, atypical carcinoids, and small cell carcinomas [2]. Intrarenal pheochromocytoma, neuroblastoma, and primitive neuroectodermal tumors may also occur [3]. They may present clinically with gross hematuria or as a mass detected on imaging studies. NE lesions of the kidney are currently classified as well- or poorly-differentiated, both being extremely uncommon [2,4]. Well-differentiated neuroendocrine tumors of renal origin are usually carcinoids, and fewer than 56 cases have been reported in the literature [5]. There is an interesting, and as yet unexplained, association of renal carcinoids with horseshoe kidneys [5-7]. The behavior of renal carinoids is not well defined owing to the small number of reported cases, and therefore prognoses are difficult to predict. Patients with advanced disease have been reported to survive for long periods of time even in the presence of tumor spread [5,8]. Progression of hepatic metastases is the predominant cause of death in patients with gastrointestinal and other cancers. For this reason the treatment of these lesions has been the focus of multiple therapeutic approaches. We report a case of a carcinoid from renal origin with a synchronous single liver metastasis on radiological studies. We will discuss different aspects of this unusual tumor, with emphasis on the treatment of liver metastases.

## Case presentation

**Figure 1:**
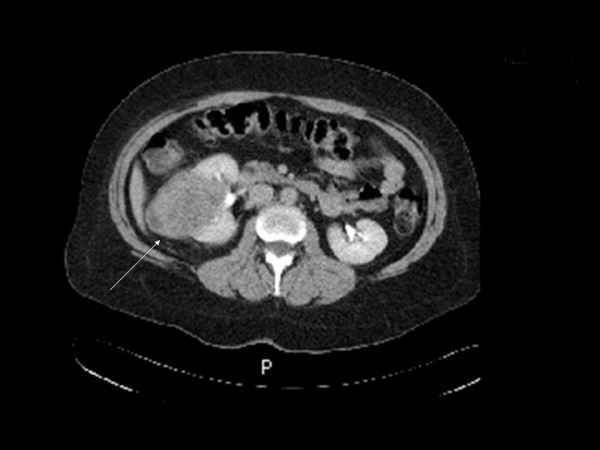
CT image showing the primary lesion in the right kidney.

**Figure 2:**
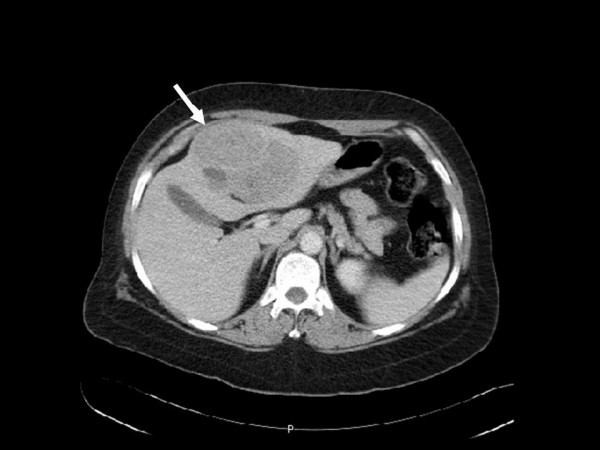
CT image showing the metastatic lesion in segments 2, 3 and 4 of the left hepatic lobe.

We evaluated a 45 year-old patient who presented initially with abdominal pain. Abdominal and pelvic CT scan showed lesions in the right ovary, right kidney, and left hepatic lobe. The right kidney mass was 8.0 cm in diameter, with areas of calcification in the periphery of the tumor inferiorly (Figure 1). In addition, there was one liver lesion, 9.7 cm in greatest diameter, located in segments 2 and 3 with extension into segment 4 of the left lobe (Figure 2). The liver and kidney tumors showed similar densities on CT scan. The right ovarian mass was multiloculated, measuring 8.7 cm in diameter. The uterus and left ovary appeared normal. CT scan of the chest showed no lesions. Tumor markers CA-125, CEA and CA 19-9 were within normal limits, as were preoperative liver function tests and renal function. A percutaneous biopsy of the liver mass was performed, which the pathologist reported to be metastatic neuroendocrine tumor. The patient was subsequently taken to the operating room, where she explored through a long midline incision; extensive abdominal examination was performed and no peritoneal seeding was found. The right ovary was removed first and sent for frozen section. Pathology reported a borderline tumor of the ovary; a total abdominal hysterectomy with bilateral salpingo-oophorectomy was performed. This was followed by a radical right nephrectomy with lymphadenectomy and a formal left hepatectomy. Final pathology reported a right ovarian borderline serous tumor, well-differentiated neuroendocrine carcinoma of the kidney (carcinoid) with 2 positive retroperitoneal lymph nodes, and a single liver metastasis. Immunohistochemistry revealed that this lesion was positive for synaptophysin and CD56, but negative for chromogranin as well as CD10, CD7, and CD20. These features are consistent with a well-differentiated neuroendocrine tumor. An octreotide scan was performed 2 months after surgery, which suggested the possibility of positive retroperitoneal lymph nodes. The patient underwent a laparoscopic left retroperitoneal para-aortic lymph node dissection, and 2 out of 5 lymph nodes were positive for tumor, with histologic features similar to the original lesions. The patient is doing well one year after her initial surgery, with no evidence of tumor recurrence.

## Discussion

Carcinoid tumors are characteristically low grade malignant neoplasms with neuroendocrine differentiation that arise in various body sites, most commonly the lung and gastrointestinal tract, but less frequently the kidneys, breasts, ovaries, testes, prostate and other locations. The prognosis of carcinoid tumors of renal origin is unclear due to the rarity of these lesions. The pathogenesis of renal carcinoid tumors (RCT) is controversial. Several hypotheses support that RCT are derived from interspersed neuroendocrine cells associated with acquired and congenital abnormalities such as metaplasia of pyelocaliceal urothelium induced by chronic inflammation, misplaced or entrapped neural crest or pancreatic tissue in the kidney during embryogenesis, activation of gene sequences common to neuroendocrine programmed cells in multipotent stem cells, or concurrent congenital abnormalities [9-12].

In 2006, an extensive review of the literature on primary RCT was published by Romero *et al*. [5]. In this report, the authors collected all previous reports by other centers for a total of 56 cases. Renal carcinoids were associated with another renal pathology in 26.8% of cases [5]. Only 7% of these patients presented with carcinoid syndrome at the time of diagnosis; interestingly, 4 other patients (7%) presented with symptoms related to other neuroendocrine syndromes. The median patient age was 49 years, with a range of 12 to 68 years. Calcifications were present on 26.5% of imaging studies. Median tumor size was 8.4 cm (range 1.5 to 30 cm) with 73.6% of patients presenting with tumors greater than 4 cm. Microscopically, 62.5% of lesions showed a mixed growth pattern with 65% demonstrating a predominant trabecular or ribbon-like growth pattern. Mitotic figures were absent or rare in 83.3% of reported cases. Immunohistochemistry demonstrated many different patterns; nevertheless, most lesions were positive for Grimelius, synaptophysin, neuron-specific enolase and chromogranin but negative for Fontana-Masson. Metastases were present in 50% of cases with para-aortic and hilar lymph nodes being the most common locations. Liver metastases occurred in 34% of cases. Metastases to the bone and spleen were also described but were much less common. Surgery was considered the treatment of choice for RCT, and long-term survival was achieved even in patients with lymph node metastases. Tumor size smaller than 4 cm at the time of diagnosis and lesions confined to the kidney were associated with a lesser incidence of metastases and better prognosis [5]. Mitotic rate was also implicated as a prognostic pathological factor.

The octreotide scan is considered the most important investigation for surveillance after resection. Following chromogranin and 5-HIAA is also recommended, even in the absence of symptoms. Additional neuroendocrine markers can be tested if they were found to be positive prior to surgery. CT and/or MRI can be used as imaging studies for surveillance. New metastases have been reported as long as 7 years after resection, indicating that long term follow up is needed.

The treatment of liver metastases from RCT is not well defined due to scarcity of cases. Most of the experience regarding the treatment of metastatic neuroendocrine disease in the liver comes from those tumors originating in the gastrointestinal tract, and in these cases, the mainstay of treatment is resection [13-15]. In our patient, an aggressive surgical approach including resection of the liver metastasis was chosen based on the biopsy results demonstrating a neuroendocrine tumor, the fact that the lesion was solitary, and that an anatomic resection could be performed to achieve negative margins. In the last few years some authors have suggested that even in the presence of extensive disease, liver resection for cytoreduction may be not only palliative, but also may increase survival [16,17]. Nagorney *et al. *[16] have proposed that surgical resection is indicated if the primary lesion is resectable or has been resected, which makes 90% of liver metastases either resectable or ablatable. An impressive 4-year survival rate of 75% has been achieved with this approach. Interestingly, they showed no survival difference between patients undergoing complete versus incomplete resection. Other reports have showed that resection of neuroendocrine tumors may achieve 5-year survival rates in the range of 47 to 92% [14,18,19], with resolution of symptoms in more that 90% and very low operative mortality.

Tumor recurrence has been a major problem after surgical treatment. Resection, ablation, or both in combination can be used to treat tumor recurrence [16]. Extensive intrahepatic recurrence can be treated with either embolization or chemoembolization, since these are usually hypervascular lesions. Systemic chemotherapy may be used in the presence of extrahepatic spread of disease [20-22]. Patients with pancreatic neuroendocrine tumors have been more responsive to chemotherapy than carcinoids. Since carcinoid lesions are low-grade, well-differentiated tumors with a low proliferation index, they are less likely to be responsive to chemotherapy. Somatostatin analogues like octreotide and more recently lanreotide, which can be given monthly, have been utilized to treat patients with advanced disease. Response rate has been variable and may correlate to octreotide scan, but stabilization of disease has been seen in 36 to 70% of patients, with a mean duration of 12 months [23]. Interferon alfa has also been used in neuroendocrine tumors with low objective response rate, but stabilization of the disease has been observed in 40 to 60% of cases [16].

## Conclusion

Early surgical intervention, together with careful surveillance and follow-up, can achieve successful long-term outcomes in patients with this rare malignancy.

## Competing interests

The authors declare that they have no competing interests.

## Authors' contributions

RG and RGR conceived of the study; RG conducted literature review and prepared the draft manuscript; RG, HJ, TDJ, PPM, and RGR performed critical editing of content and helped in preparation of the manuscript; RG and DR edited the final version. All authors read and approved of the final version of the manuscript
